# Duties of Physicians as Examiners for Life Insurance1Read before the sixteenth annual convention of the Mutual Life Insurance under-writers, at Minneapolis, Minn., June 16, 1891.

**Published:** 1891-09

**Authors:** George L. Brown

**Affiliations:** Medical Director of the Masonic Life Association of Western New York; 173 Niagara Street


### DUTIES OF PHYSICIANS AS EXAMINERS FOR LIFE INSURANCE.[1]

By GEORGE L. BROWN, M. D.,

Medical Director of the Masonic Life Association of Western New York.

History informs us that the plan of life insurance had its origin in England in the sixteenth century. It began with what were called friendly societies, and its beginnings were, of course, crude and experimental. At first the payment only of small sums for burial expenses, or for annuities to widows of clergymen, was attempted. Benevolent societies, with similar objects, which have been enlarged from time to time, have existed in America almost from the beginning of its settlement. Their growth has been marvelous, and the good they have accomplished, and are still effecting, is incalculable.

But the failures have been many, and have very clearly demonstrated that success can be had only by adherence to business principles. To be successful, the assessment or premium must be carefully adjusted to the risk, and the risk cannot be intelligently estimated without scientific investigation. Low assessments are possible only with low mortality, and it is only with low assessments that the coöperative associations can compete successfully with innumerable other companies that are seeking business.

Until quite recently applicants were received as a matter of charity, or fraternity, and without medical examination. But it very soon appeared that the losses were so heavy that the survivors could not and would not meet the necessary assessments. Good fellowship, mutual aid and sociability are well in their place, but will not, nor should they, make an applicant for insurance pay more for it than it is worth. It becoming clear that the existence

1. Read before the sixteenth annual convention of the Mutual Life Insurance Underwriters, at Minneapolis, Minn., June 16, 1891.

and prosperity of the associations depended almost entirely on the contingencies of health and life, physicians were called upon to make such examinations as should detect the diseased and unhealthy, and it has clearly appeared that their services are indispensable.

Their duties are delicate and important. A single mistake from careless or insufficient examination may cost the assessment payers thousands of dollars. And it must be confessed that such mistakes are not uncommon. That is but too clearly apparent from our mortuary tables. Among the causes of death of persons who have been, only a few months before the deaths occur, accepted by the most skilful and careful physicians, we find consumption, organic disease of the heart, cirrhosis of liver, uremic poisoning, diabetes, cancer of the various organs, cystitis nephritis, Addison's disease, etc. Great injustice has been done, and daily is done, to the associations and companies, and to applicants, by the hurried and insufficient manner in which examinations are made. That such should be the case is doubtless a reproach to our profession, but we think the responsibility for it lies principally upon the management of the companies. Much too often they are willing neither to pay a fair price for the thorough examination of applicants, nor afford suitable opportunities and facilities for making it.

The great competition that exists among all classes of life insurers has led to very unfortunate and undesirable practices. Not only the coöperative, but both the stock and mutual associations, expect and require their doctors to visit an applicant at his factory, store, counting-room, or residence, and, if fortunate enough to find him, to undertake the duties of a canvasser, and argue the claims and merits of their respective schemes. If successful in convincing him of them, he is then and there to make his professional examination, in the presence of others and amidst the noise and confusion of rattling machinery, musical instruments, type-writers, or children.

And not infrequently he is called upon in the lodge room to examine a large number of charter members in a single evening, and in the presence of each other. How does, and under such circumstances, how must he perform his work? By counting the pulse, looking at the tongue, taking the measurements and listening to the heart and lungs through ordinary clothing. And he is paid for the examination that he makes, the sum of from one to five dollars, according to the fixed rules of the association or company. Is it not apparent that such investigation is necessarily superficial and insufficient, and that in such practices is to be found the reason why the death losses are, in many of our associations, so heavy and so out of due proportion to the assessments that have been expected to suffice? The matter is one of sufficient importance to call for the most careful and thorough exercise of professional skill. Nothing less than that should be accepted, and the payment made should be commensurate with the value of service required. The physician should practice what he has been taught,—"to see with his own eyes, feel with his own hands, and hear with his own ears." In justice as well to the applicant and the company as to himself and his own professional reputation, he should conduct his examinations only in his own office. The applicant should disrobe. His exact height and weight should be taken, and their proportion to each other accurately noted. Thorough investigation should be made as to indications of dietic, parasitic, diathetic, and genetic diseases.

Very many diseases of the chest and abdomen, acute as well as chronic, can be detected by changes in the color of the skin from its normal appearance. It has been truly said that a man who has a perfectly healthy skin is almost certain to be healthy in other respects. The examiner should observe whether the skin be dry or moist and clammy, pale, blanched or livid, jaundiced, or of that dull, earthy hue which at once gives the impression of serious organic disease, associated with disordered nutrition and emaciation, cyanosis, emphysematous or sclerotic affections.

The general character of the constitution, whether vigorous or otherwise, should be carefully noted. Particular attention should be given to the form and development of the chest, whether enlarged and bulging, or contracted and depressed in particular places. The form and position of the sternum, clavicle and ribs should be noted, and traces looked for of pleuritic effusion or absorption of pleuritic exudation, or of caseous degeneration or tuberculosis of the lungs, and search made for indication or vestiges of rickets and diseases of the vertebral column. The movement of respiration, its extent and frequency, must be watched, and a complete inspection and palpation of the abdomen will be of vast importance in many cases. The temperature of the body must be duly recorded, and as in healthy persons that is subject to daily fluctuation, rising continually from morning to evening, and sinking again until morning, the hour of the day when the examination is made should be noted.

Exercise, according to its violence and duration, raises temperature; the taking of food and drinks has a similar effect which should not be overlooked, but carefully estimated. In examining the lungs, heart, and liver, the physician cannot exercise too much care. Many cases of pulmonary tuberculosis in its incipient stage can be detected by cautious scrutiny, in which one is much assisted by certain marked characteristics, such as large eyes, long eye-lids, thin slender hands, curved nails and leanness of the chest, that indicate a predisposition to the disease, when it cannot be discovered by other means. The sounds of the heart and general phenomena of the circulation should be carefully examined and recorded. The liver and the condition of the primæ viæ and digestive system should be accurately examined. The urine, which is the product of eliminative action in the body, should always be voided in the presence of the examiner, and the applicant can usually succeed in inducing a flow of it by immersing his hands in cold water. It should be analyzed before it has stood long enough to undergo a chemical change. And here the question arises, How far should the examiner proceed with his analysis? At present our companies require only tests for albumin and sugar, provided the specific gravity and color are nearly normal and the reaction acid. If there is albumin or sugar present, the risk is generally disapproved. But it is very questionable whether that is just in every case. It is well understood that shortly after one has partaken freely of highly albuminous food, albumin is to be found in his urine, although his health is perfect. The same is true as to sugar after an excess of saccharine food. Here permit me to quote from Henry S. Millard, M. D.: "Should all cases of chronic albuminaria, simply from the fact of albumin being found, be rejected by life insurance examiners?" He says they should not, "as he has known some albuminuric patients who enjoyed practically good health, and lived to a good old age." "Albuminuria is not always a more threatening symptom than other symptoms; an albuminuric patient may occasionally be in every respect a good risk." "There are cases where repeated and comprehensive examinations must be made before the examiner can decide as to the interests of his company." There can be no unvarying rule as to this point. He has known albuminuric subjects rejected whose health was good, and applicants with other affections much more serious than some of these cases accepted.

I have myself known persons with sugar in their urine who have enjoyed good health and outlived their expectancy, according to our expectancy tables, and still they could not be accepted as fair risks under our rules. I believe that the examiner should proceed further than those rules direct, and make analysis for bile pigments, corpuscles and epithelial casts. It is highly important that in the examination of every applicant, some blood should be drawn and submitted to careful scrutiny and to microscopical inspection. The color and number of both the red and the white corpuscles, and the proportion as well as the character of the constituents of the fluid should be noted. Almost all diseases of the rectum and anus are called "piles" by the majority of people. How many of the physicians of life insurance companies ever examine an applicant to ascertain whether he is afflicted with piles, fistula, pruritus, polypi, fissures, warts, ulcers, prolapsus, proctitis, or cancer of the rectum? But to fail to do so is to commit a great injustice.

It appears to me to be very clear that the examining physician of every assurance society, or company, should be expected and required to examine all applicants in a private, quiet place, where he can have the convenient use of all necessary instruments and appliances. He should have at hand a tape line, fever thermometer, stethoscope, stethometer, spirometer, cyrtometer, a percusser and pleximeter, tuning fork, cardiometer sphygmograph, tongue depresser, urethral sounds, rectum and ear speculums, microscope, urinometer, test tubes, re-agents and pipettes, litmus paper, laryngoscope, throat mirror, and scales.

A thorough examination and record should be made in regard to the family history, resemblances and predispositions, habits of life, occupation and nationality of those who are connected by ties of consanguinity, or marital relations with the applicant; his own habits in regard to the use of stimulants, narcotics, tobacco and patent medicines, should be noted. His general appearance, the scars of previous vaccinations, the history of all diseases that he has suffered from, scars, defects and special marks, his peculiarities as to having large or small bones, his temperament, the color of his hair and eyes, and everything pertaining to his health and the prospect for it, should go into the record. And it is important that the record be made by the examiner, in his own handwriting, and not, as is now customary, by a solicitor or agent. If I seem to you to have outlined a pretty wide field of professional duty for the medical examiner, I can only ask, What part of it can with safety be omitted?

Our associations, to succeed, must strictly adhere to the business principle of maintaining only such mortality rates as our assessments will justify, and our assessments are limited by competition. The theory that we are simply charitable institutions and exempt from the restraints of business rules, has necessarily been abandoned. If it had not been, all medical examinations should either be dispensed with or reversed so that their aim should be to discover the most diseased and precarious cases, as such are certainly the most needy objects for charity. If we are to have any scientific scrutiny of applications, let us have something that will give a firm basis for intelligent judgment. Anything short of that is a reproach to the medical fraternity and dangerous to the association. To save the loss occasioned by improper acceptance of a single application, followed in a few months by a death that results from a discoverable disease, is to have the cost of many such examinations as have been described? And, on the other hand, they might prove that cases now rejected are perfectly good, and our associations cannot be maintained without the constant influx of good cases.

173 Niagara Street.

